# The mechanism of peroxisome motility in filamentous fungi

**DOI:** 10.1016/j.fgb.2016.10.006

**Published:** 2016-12

**Authors:** Gero Steinberg

**Affiliations:** School of Biosciences, University of Exeter, Stocker Road, Exeter EX4 4QD, United Kingdom; Donders Chair, University of Utrecht, Department of Biology, Padualaan 8, 3584 CH Utrecht, The Netherlands

**Keywords:** PO, peroxisome, EE, early endosomes, LD, lipid droplets, MT, microtubules, Organelles, Early endosomes, Organelle transport, Active diffusion

## Abstract

Peroxisomes (POs) are an essential part of the fungal cell’s inventory. They are involved in cellular lipid homeostasis, reactive oxygen metabolism and in the synthesis of secondary metabolites. These functions are thought to require frequent organelle-organelle interactions and the even-distribution of POs in fungal hypha. Recent work in the basidiomycete *Ustilago maydis* and the ascomycete *Aspergillus nidulans* reveals a crucial role of early endosomes (EEs) in the dynamic behavior of POs required for their cellular distribution and interaction. This article summarizes the main findings, which provided unexpected insight into the mechanism by which fungal cells organize themselves.

## Highlights

• Early endosomes transiently bind peroxisomes and move along microtubules in the hyphal cell.
• Bi-directional motility of early endosome causes turbulences within the cytoplasm, which enhances diffusion of peroxisomes.
• Both mechanisms cooperate to distribute peroxisomes, as well as lipid droplets in hyphae.

## 1 Introduction

Peroxisomes and lipid droplets participate in the metabolism of reactive oxygen species and beta-oxidation of fatty acids, but also the synthesis of various secondary metabolites, including antibiotics, biotin and the toxin paxilline and AK-toxin (overview in van der Klei and Veenhuis, 2013). In filamentous fungi, only a small portion (∼5%) of POs undergo rapid and directed transport, whereas the vast majority show diffusive, non-directed motility (Guimaraes et al., 2015, Lin et al., 2016; Video 1). This rapid and directed motility of POs is reminiscent of moving fungal early endosomes (EEs), which were discovered in *U. maydis* (Wedlich-Söldner et al., 2000), but subsequently also described in other fungi (e.g. Abenza et al., 2009, overview in Steinberg, 2014). Most recently, studies in both *U. maydis* and *A. nidulans* demonstrate that kinesin-3 and dynein (which drive EE motility, Steinberg, 2014), bind directly to EEs via an endosomal hook-adapter complex (Bielska et al., 2014b, Zhang et al., 2014, Yao et al., 2014). Such direct interaction between motors and adapters suggested that there are PO-specific motors and adapters. However, recent work in *U. maydis* (Guimaraes et al., 2015) and *A. nidulans* (Salogiannis et al., 2016) revealed a fundamentally different mechanism by which POs undergo directed transport in fungal hyphae, where motors bind to EEs that indirectly move POs.

Video 1Motility of POs in the basidiomycete fungus *U. maydis*. Most POs show random short-range and non-directed motions, whereas a few POs undergo long-range and directed motility. The cell edge is indicated in blue, POs were labeled with GFP-SKL (Guimaraes et al., 2015). Time is given in seconds and milliseconds. Scale bar is 10 μm.
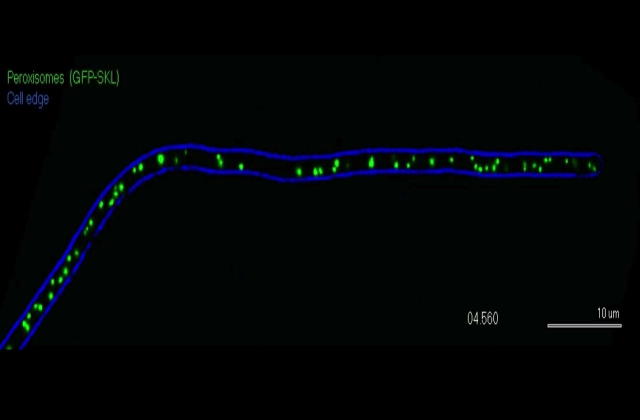

## 2 Peroxisomes hitchhike on early endosomes

It is widely-accepted that motor proteins bind directly to their cargo to move it along the fibers of the cytoskeleton. Studies in *U. maydis* showed that kinesin-3 is essential for motility of POs towards the plus-end of MTs, which strongly suggested that kinesin-3 binds to POs (Guimaraes et al., 2015). However, when Guimarares and co-workers tried to co-localize kinesin-3 and POs, they found that kinesin-3 does not co-migrate with POs, but instead it localizes ∼400 nm ahead of the moving POs. Kinesin-3 moves EEs (overview in Steinberg, 2014), suggesting that EEs are involved in PO motility. Indeed, when the authors co-observed POs and EEs, they confirmed that PO and EE were moving in pairs, with EEs leading (Video 2; EEs labelled by the hook adapter protein Hok1, Bielska et al., 2014b). Subsequent mutant studies revealed that abolishing EE motility stopped PO transport, suggesting that EEs drag POs throughout the fungal cell. A similar mechanism underlies motility of lipid droplets (LDs), but to a lesser degree also the movement of endoplasmic reticulum tubules (Guimaraes et al., 2015), suggesting that various organelles “hitchhike” on moving EEs. Most recently, PO “hitchhiking” on EEs was also described in *A. nidulans* (Salogiannis et al., 2016). This suggests that this unusual transport mechanism is of general importance in filamentous fungi. While such mechanism is not yet reported in animal cells, the principle of “hitchhiking” in intracellular transport is conserved (Salogiannis and Reck-Peterson, 2016). Studies in *A. nidulans* also identified an EE-bound adapter protein, PxdA, which was required for PO-EE interaction in *A. nidulans* (Salogiannis et al., 2016). PxdA orthologues are present in other ascomycetes (*Magnaporthe oryzae*, *P* = 9.0e−38, NCBI accession number: EAQ71055.1; *Neurospora crassa*, *P* = 1e−30, NCBI accession number: XP964921.21), but is not present in the genome of *U. maydis*. Thus the molecular mechanism of this organelle-organelle interaction remains to be elucidated. Interestingly, the interaction between EEs and POs, as well as EEs and LDs in *U. maydis* was transient. In other words, POs and LDs bind to EEs, move a certain distance and then fall off the moving organelles. Such binding and dissociation was previously described for polysomes (ribosomes and mRNA), which also hitchhike on moving EEs in *U. maydis* (Higuchi et al., 2014, Baumann et al., 2014). Thus, transient interaction with EEs enables directed transport of other organelles, including POs and LDs, but also the cellular machinery for protein synthesis.

Video 2Co-motility of POs (red) and EEs (green) in the basidiomycete fungus *U. maydis*. A pair of a PO, labelled with the marker protein mCherry-SKL, and an EE, labelled with the hook adapter Hok1-GFP (Bielska et al., 2014a, Bielska et al., 2014b) moves towards the hyphal tip (indicated by arrow and “Tip”). Note that the PO “waves” behind the leading EE. Also note that PO motility stops when the EE dissociates (>3.0 s, last 4 frames of video sequence). Time is given in seconds and milliseconds. Scale bar is 1 μm.
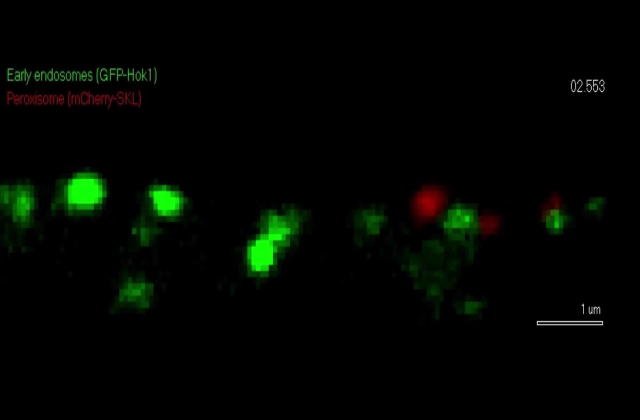

## 3 Early endosomes underlie active diffusion of fungal organelles

The majority of fungal POs and LDs do not undergo directed motility, but instead undergo short-range and undirected motions (Guimaraes et al., 2015, Lin et al., 2016; Video 1). Such random movements are reminiscent of Brownian motion, which is due to thermal collision with water molecules and therefore considered a passive diffusive process. However, a recent study in *U. maydis* demonstrated that random motion of POs and LDs requires enzymatic activity (Lin et al., 2016). This is best illustrated when in the presence of the respiration chain inhibitor carbonyl cyanide m-chlorophenylhydrazone (CCCP), which lowers cellular ATP levels and inhibits diffusive motility of POs. The phenomenon that random and non-directed motions within the cell depend on enzyme activity is known as “active diffusion” (Brangwynne et al., 2009). In *U. maydis*, active diffusion of POs is due to MT-dependent rapid bi-directional EE motility. This mechanism is not identical with the transient interaction of POs with EEs (see above), as it is slower and non-directed. In fact, EEs motility is likely to cause turbulences within the crowded cytoplasm, which indirectly enhance the diffusion of POs. Mathematical modeling of this process revealed that active diffusion and directed transport co-operate in two ways (Lin et al., 2016). Firstly, both oppose a slow myosin-5-based drift of POs towards the hyphal tip, and this most likely reflects constant delivery of secretory vesicles towards the growing hyphal apex. Secondly, both active diffusion and directed transport of POs is required for effective mixing of the PO compartment, which allows efficient organelle-organelle interactions typical for POs and LDs (Lin et al., 2016). Currently, this mechanism is only described in *U. maydis*. However, EEs show rapid and constant bi-directional motility in ascomycetes (Abenza et al., 2009), which suggests a similar role of these organelles in active diffusion of POs and LDs in these fungi.

Video 3Schematic summary of the role of EEs in directed transport and active diffusion of POs. Note that only dynein-dependent EE motility to minus-ends of MTs is shown; similar processes occur when EEs are transported towards plus-ends by the motor kinesin-3. EE motility underlies the dynamic behavior of POs in 2 ways: (1) EEs bind to POs via a specific adapter and drag POs throughout the cytoplasm, (2) moving EEs cause turbulences in the cytoplasm (indicated by spiral arrows), which enhances diffusion of POs. Note an PO adapter was identified in *A. nidulans* (PxdA, Salogiannis et al., 2016) and has orthologues in other ascomycetes, but is not present in the genome of *U. maydis*.
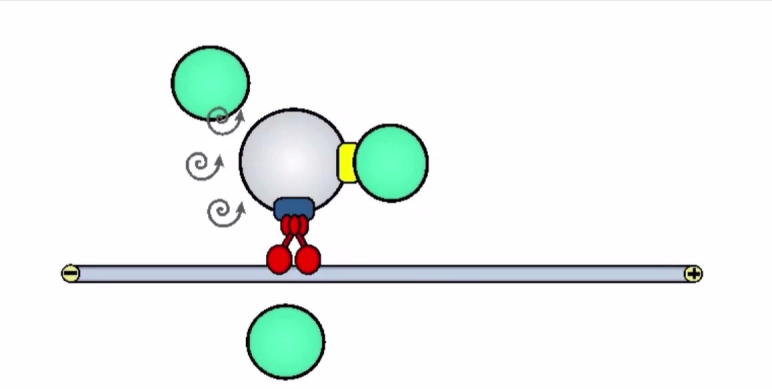

## 4 Conclusion

In this focused Video article, I have reviewed recent work that demonstrated that EEs mediate directed transport of POs in fungi. Surprisingly, work in *U. maydis* showed that POs, but also LDs, transiently interact with moving EEs, thereby “hitchhiking” for long-range directed motility in the hyphal cell (Guimaraes et al., 2015). This mechanism was also found in *A. nidulans*, where a specific adapter links POs to EEs (Salogiannis et al., 2016). In addition, constant EE motility enhances the diffusive motions of POs, required for local mixing and organelle-organelle interactions in *U. maydis* (Lin et al., 2016). These results demonstrate that EEs mediate PO motility in two ways, (1) by direct interaction, which drags POs over long distance through the cell (Video 2), and (2) causing cytoplasmic turbulences that indirectly increase PO diffusion (Video 3). Both roles are also found for LDs. These findings argue for a central role of EEs in spatially organizing organelles in filamentous fungi. Such essential cellular role adds to the increasing repertoire of functions for EEs in filamentous fungi. These include long-range signaling (Bielska et al., 2014a) and distribution of mRNA and ribosomes (Baumann et al., 2014, Higuchi et al., 2014; overview in Steinberg, 2014). Thus, from the initial discovery of EEs some 16 years ago, intensive research in various fungi has provided an increasingly detailed insight into the molecular machinery of EE transport and has revealed important aspects of their function. Whilst more discoveries are to be expected, EEs have emerged as master organizers of the fungal cell.
