# Supplementary figures and images for: The mechanism of peroxisome motility in filamentous fungi
Source: Fungal Genet Biol. 2016 Dec;97:33–5. doi: 10.1016/j.fgb.2016.10.006 (PMC5176036; doi:10.1016/j.fgb.2016.10.006)

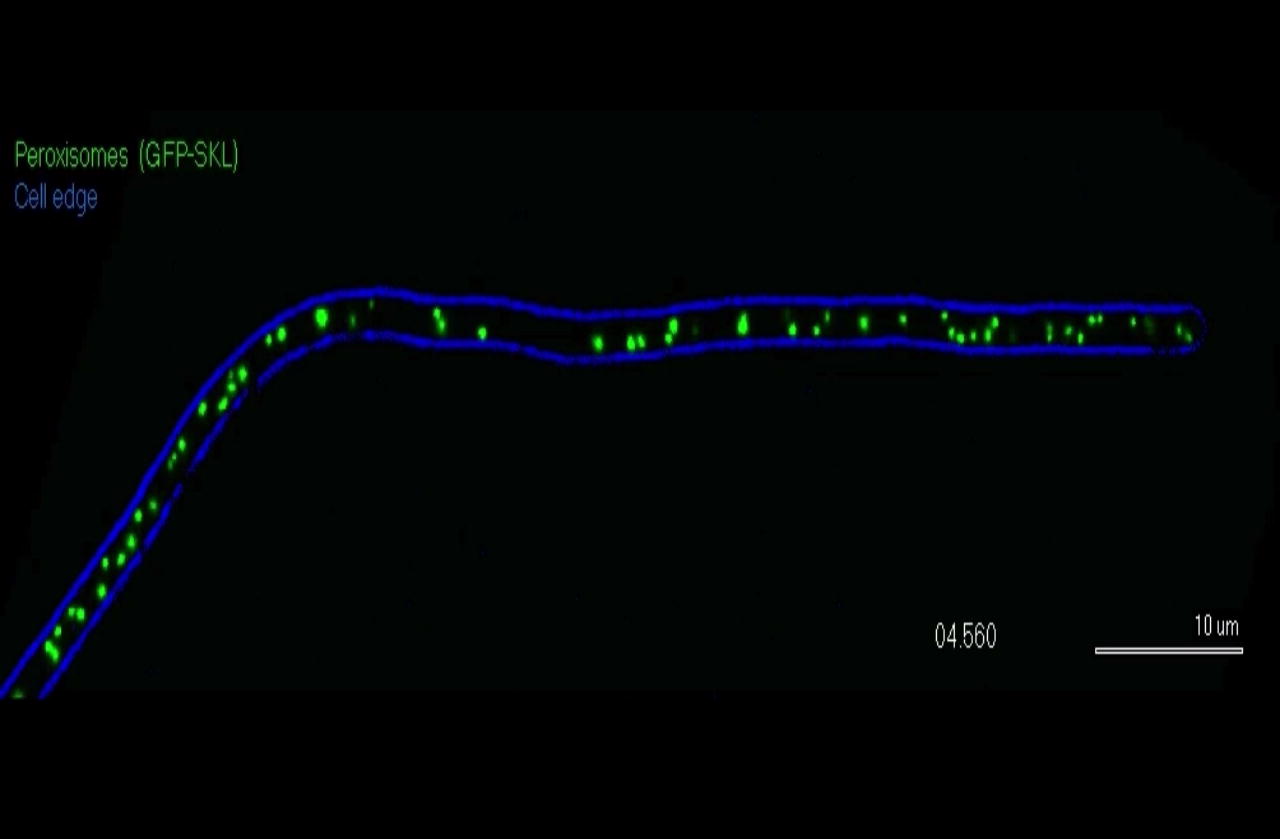

Supplement: Video 1 — Motility of POs in the basidiomycete fungus U. maydis. Most POs show random short-range and non-directed motions, whereas a few POs undergo long-range and directed motility. The cell edge is indicated in blue, POs were labeled with GFP-SKL (Guimaraes et al., 2015). Time is given in seconds and milliseconds. Scale bar is 10 μm. [file mmc1.jpg]

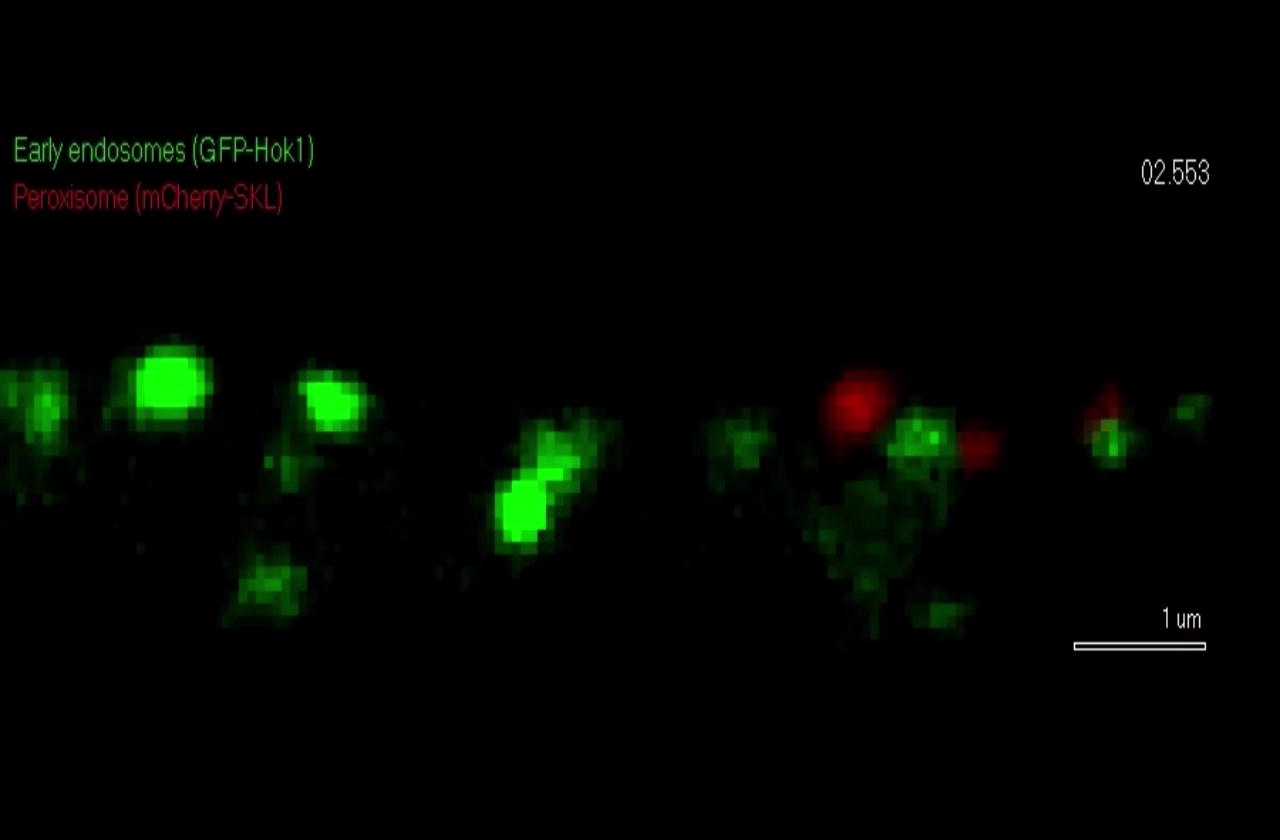

Supplement: Video 2 — Co-motility of POs (red) and EEs (green) in the basidiomycete fungus U. maydis. A pair of a PO, labelled with the marker protein mCherry-SKL, and an EE, labelled with the hook adapter Hok1-GFP (Bielska et al., 2014a, Bielska et al., 2014b) moves towards the hyphal tip (indicated by arrow and “Tip”). Note that the PO “waves” behind the leading EE. Also note that PO motility stops when the EE dissociates (>3.0 s, last 4 frames of video sequence). Time is given in seconds and milliseconds. Scale bar is 1 μm. [file mmc2.jpg]

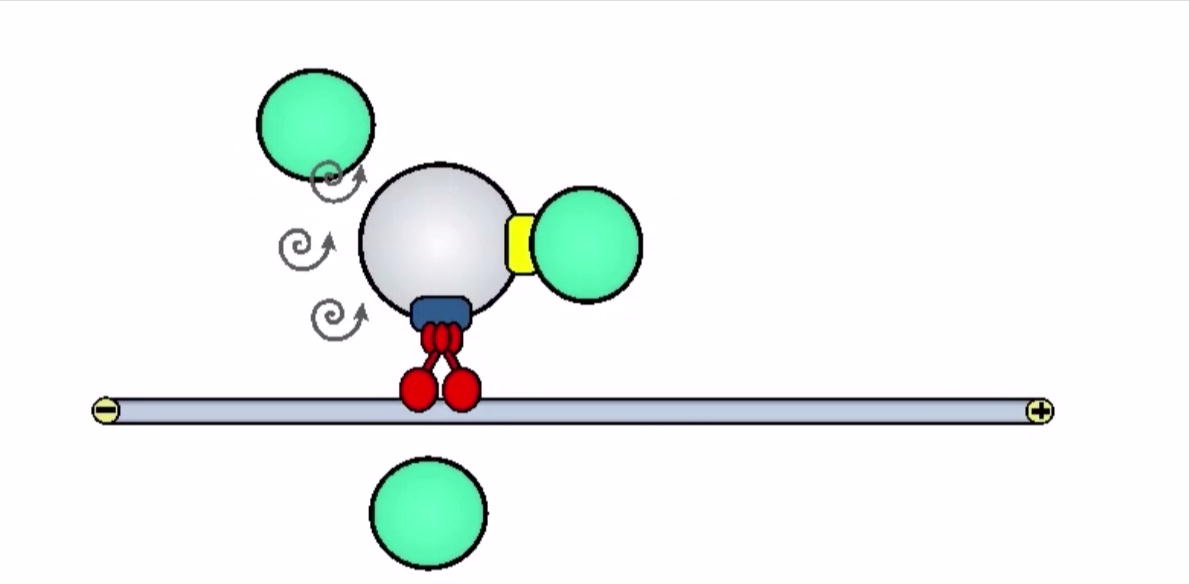

Supplement: Video 3 — Schematic summary of the role of EEs in directed transport and active diffusion of POs. Note that only dynein-dependent EE motility to minus-ends of MTs is shown; similar processes occur when EEs are transported towards plus-ends by the motor kinesin-3. EE motility underlies the dynamic behavior of POs in 2 ways: (1) EEs bind to POs via a specific adapter and drag POs throughout the cytoplasm, (2) moving EEs cause turbulences in the cytoplasm (indicated by spiral arrows), which enhances diffusion of POs. Note an PO adapter was identified in A. nidulans (PxdA, Salogiannis et al., 2016) and has orthologues in other ascomycetes, but is not present in the genome of U. maydis. [file mmc3.jpg]
